# Anchote (*Coccinia abyssinica [Lam.] Cogn.*) powder, an underutilized indigenous crop, as a substitute to commercial pectin in the production of strawberry jam

**DOI:** 10.1016/j.heliyon.2022.e10700

**Published:** 2022-09-18

**Authors:** Adugna Mosissa Bikila, Yetenayet Bekele Tola, Tarekegn Berhanu Esho, Sirawdink Fikreyesus Forsido

**Affiliations:** a Department of Post-Harvest Management, College of Agriculture and Veterinary Medicine, Jimma University, P. O. Box 307, Jimma, Ethiopia; b Department of Food Science and Nutrition, Faculty of Agriculture, Wollega University, P. O. Box 38, Shambu, Ethiopia; c Department of Industrial Chemistry, Addis Ababa Science and Technology University, P. O. Box 16417, Addis Ababa, Ethiopia

**Keywords:** Anchote powder, Jam, Pectin, Physicochemical properties, Texture

## Abstract

The study investigated the potential of anchote (*Coccinia abyssinica [Lam.] Cogn.*) tuber powder as a substitute to commercial pectin. Mixture D-optimal design was used to generate 14 experimental runs using ranges: strawberry fruit (45–55%), sugar (43–53%), and anchote powder (0.75–1.75%). The effect of anchote powder on physicochemical and textural qualities of the jams was evaluated. The parameters measured include: moisture content (30.7–32.8%), total soluble solid (50.7–65.4 ºBrix), water activity (0.73–0.80), pH (2.93–3.13), titratable acidity (0.58–0.72%), gel strength (326.39–440.37 g mm), hardness (26.36–35.09 g), cohesiveness (0.89–0.94), energy of penetration (418.72–489.51 g s), adhesiveness (−25.38 to −103.79 g s) and stickiness (−13.78 to −29.22 g). The jam formulation J_13_ (50% strawberry, 48.2% sugar, 1.33% anchote) was best performing. Numerical optimization showed the best combination of parameters at 52.4% strawberry, 46.0% sugar, 1.07% anchote. The jam formulated with anchote powder was comparable with the jam made using pectin.

## 1 Introduction

**Figure 1:**
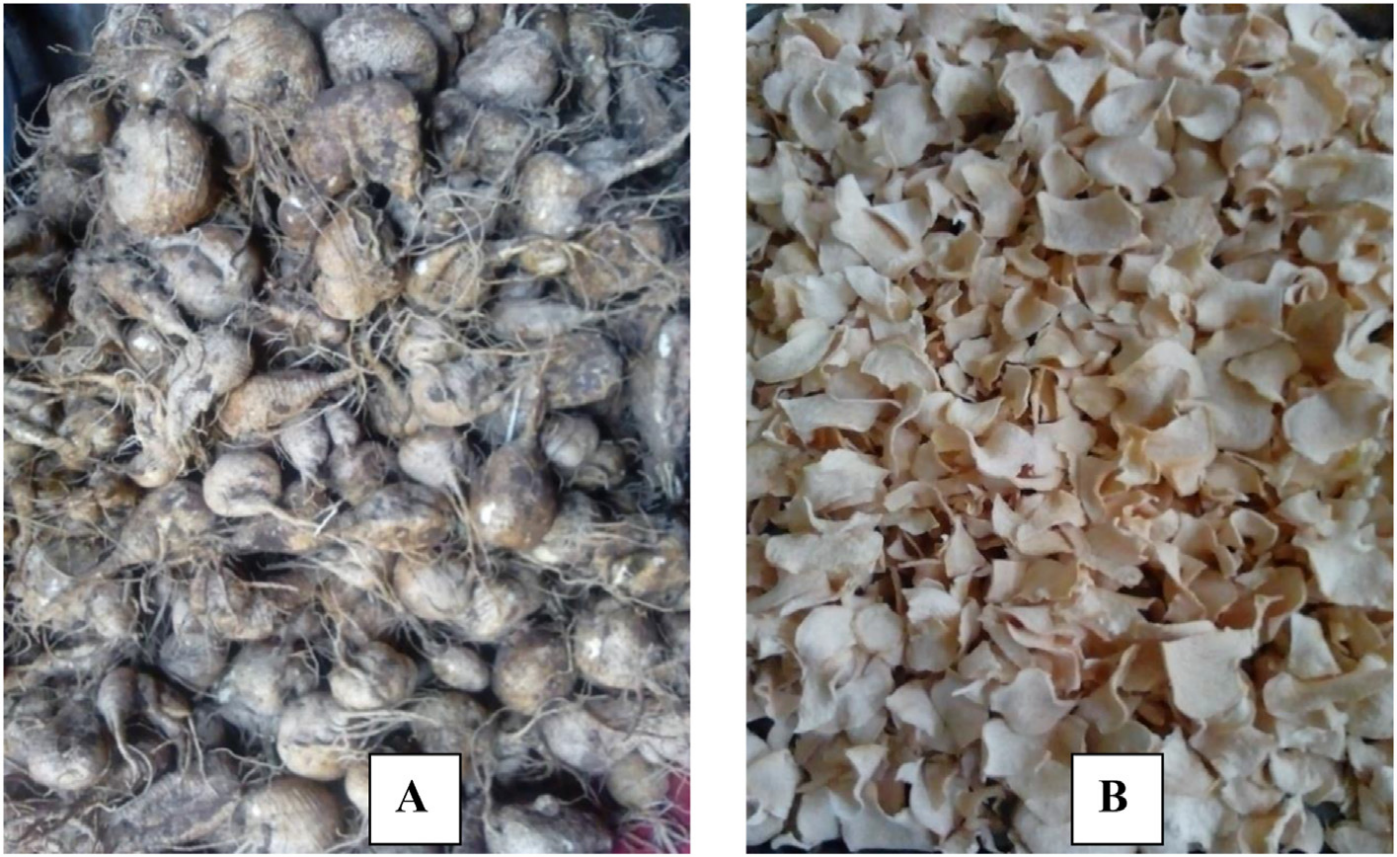
**A-**Fresh anchote tuber, **B-**dried anchote tuber slices.

Anchote (*Coccinia abyssinica [Lam.] Cogn.*) is one of the underutilized food crops that originate in Ethiopia, particularly in Wollega Zones of the Oromia Region (Fekadu et al., 2014). The most common edible part of anchote is its tuber (Figure 1), though its leaf and young fruit are also consumed in some areas (Ayalew, 2016). The crop is usually harvested after 3–5 months of planting (Abera and Haile, 2015; Mekbib and Deressa, 2016), with an estimated 15–18 tons/hectare yield (Fekadu et al., 2014). From a nutrition point of view, the tuber contains carbohydrates (80.0–83.7%), proteins (3.8–4.5%), fibers (7.5–8.2%) and ash (4.6–4.7%) in its dry form (Bikila et al., 2020). Its nutrient content is relatively higher than other root and tuber crops (Abera and Haile, 2015), especially unique in its high protein and calcium. In addition to this, the crop has medicinal, economic and social importance in the community (Ayalew, 2016; Yambo and Feyissa, 2013). The tuber is traditionally processed by boiling and sometimes further cooked before consumption (Gemede, 2014).

In the Wollega area of Ethiopia, anchote stew (locally called *“ittoo ancootee”*) is a common processed product available with injera (*Ethiopian soft bread-like fermented product made from Teff*) in restaurants (Aga and Badada, 1997; Parmar et al., 2017). However, it is not processed and used commercially to produce value-added products and additives in food industries. Recently, attempts were made to acknowledge the advantages of the crop regarding its agronomic performance and nutritional value. Research work indicated that the tuber could be used as a food ingredient in commercially produced shelf-stable products due to its relatively higher calcium and protein contents than other tuber crops (Ayalew, 2016). As a result of its high calcium content, the crop is traditionally recommended for people suffering from bone fractures or displaced joints, and for rapid recovery and strength of lactating mothers (Parmar et al., 2017).

Some important parameters of processed anchote (*C. abyssinica*) tuber flour are indicated in our previous works (Bikila et al., 2020, 2021, 2022). However, there have been no attempts to use anchote powder as an ingredient to produce industrial-based shelf-stable products. The powder could be one of the potential plant products to complement the function of pectin as a stabilizer and texture modifier in the production of jams and jellies. In this regard, some studies showed that powders obtained from plant seeds could be used as a substitute for commercial pectin in jam production (Ndabikunze et al., 2011). The potential use of anchote for this purpose can be emanated from the high calcium and amylopectin content of its powder which could assist in creating a cross-link and strong network to stabilize the textural properties of jam and jellies. Calcium is also a recommended ingredient as a firming agent in jam and jelly preparation by Codex Alimentarius Commission (CODEX, 1981). Jam is a popular commercial product that is usually made from different fruits, vegetables or mixture of them (Awulachew, 2021; Korus et al., 2017). There is also recent initiation to promote processing of perishable fruits into jam in Ethiopia (Tadele et al., 2022).

Fruits with low pectin content, such as strawberries (*Fragaria x ananassa*), need the addition of commercial pectin for jam and jelly formation (Islam et al., 2012). Pectin is a sugar/polysaccharide compound that makes jam soft but thick in texture (Nissa et al., 2019). It helps to jellify a fruit-based jam at the proper level of pH and concentration of sugar. Jam properties are developed by the interaction of pectic substances, the pulp of the fruit, sugar volume and pH of the jam. Particularly, the gel structure and texture is determined by pectin concentration which may range from 0.5 to 1.5% by weight for commercial jam manufacturing (Shahnawaz and Shiekh, 2011). However, commercial pectin is not commonly available in stores in developing countries. In addition to this, as an imported product, the price is high and inflates the cost of production of jams and jellies. A low-cost, locally available gelling agent is recommended for sustainable production and supply of jams which will replace the function of commercial pectin. In this regard, the powder from anchote is one of the potential substitutes to replace the function of commercial pectin. However, the gelling potential of anchote powder is unfamiliar to both small and large scale food processors due to limited scientific information.

Therefore, the study aimed to investigate the possible use of anchote powder as a gelling agent alternative to commercial pectin in the preparation of jam from strawberry (*Fragaria x ananassa*) fruit. Strawberry is nutritionally important fruit, but it is highly perishable and its pectin content is low (Islam et al., 2012; Sicari et al., 2020). The fruit is commonly processed into jam for preservation and commercialization. Hence, strawberry fruit is purposively used to evaluate the gelling property of anchote powder. The use of anchote powder as an ingredient in a jam gelling technology could support the production of jams with different ingredients and develop an anchote value chain as a commercial product to the food industry.

## 2 Materials and methods

### 2.1 Raw materials

Strawberry (*Fragaria × ananassa*) fruit, purchased from the local market, was selected for jam preparation to evaluate the gelling efficiency of anchote powder. Strawberry fruit is chosen because of its low pectin content compared to other fruits and its wide use in jam production (Islam et al., 2012). Table sugar and food-grade 5% acetic acid (*Puro Aceto Vinegar (ZAT), Ethiopia*) were purchased from the local market as a sugar source and to modify the jam pH. Commercial pectin made from natural fruit peel (*crystals, U.S.A*) was used as a positive control. Blanched and oven dried anchote (*C. abyssinica*), *Desta 01* variety, powder was used for the study as a commercial pectin substitute.

### 2.2 Anchote powder preparation

Fresh anchote tuber was obtained from Debrezeit Agricultural Research Center at maturity level of 3½ months of planting in December during which the average temperature was 10–16 °C. Then, the tuber was washed in running tap water to remove the adhering substances, peeled off using stainless steel knives, sliced to about ∼2 mm thickness, and immediately immersed in water before blanching. Blanching was accomplished in boiling water (98 ± 2 °C) for 5 min to avoid enzymatic browning; and dried in a hot air oven (*LABQUIP, LEICESTER LE67* 5FT*, England*) at 60 °C to constant weight based on the results of the previous study for better thickening and gelling capacity (Bikila et al., 2022). The dried slices were ground into flour, sieved through a 500 *μ*m mesh, packed in a moisture-proof polyethylene bag and stored at −4 °C until use.

### 2.3 Experimental design

The quality of jam is mainly determined by the proportions of sugar and pectin added to the fruit pulp (Disha et al., 2017). Three factors (ratio of fruit, sugar and gelling agent) were considered in this study to optimize strawberry jam with an anchote powder substituting pectin. In most cases, a fruit pulp to sugar ratio of 1:1 was used for quality jam preparation with a fruit content not less than 45% (FAO, 2009). Design-Expert 13 Mixture D-optimal design was used to develop the proportion of the three ingredients (Table 1). The software generated fourteen runs of the experimental matrix as indicated in Table 1, using a range from 45–55%, 43–53% and 0.75–1.75% for strawberry fruit, sugar and gelling agent (anchote powder), respectively. The acid content was kept constant in all proportions to maintain the desired pH range in strawberry Jam making.

**Table 1:** Proportion of strawberry fruit pulp, sugar, anchote powder and acid proportions (%) as designed by Design expert software.

Treatments	X_1_	X_2_	X_3_	AC
**J1**	45.8	53.0	0.75	0.5
**J2**	45.0	52.8	1.75	0.5
**J3**	45.8	53.0	0.75	0.5
**J4**	52.5	45.9	1.04	0.5
**J5**	55.0	43.8	0.75	0.5
**J6**	54.8	43.0	1.75	0.5
**J7**	55.0	43.8	0.75	0.5
**J8**	47.9	50.5	1.04	0.5
**J9**	45.0	52.8	1.75	0.5
**J10**	54.8	43.0	1.75	0.5
**J11**	49.9	47.9	1.75	0.5
**J12**	52.4	45.5	1.54	0.5
**J13**	50.0	48.2	1.33	0.5
**J14**	50.4	48.4	0.75	0.5
**Cont1**	49.7	49.7	0.20	0.5
**Cont2**	49.8	49.6	0.00	0.5

X_1_ = Starawberry fruit pulp (%), X_2_ = Commercial sugar (%), X_3_ = Anchote tuber powder (%), AC = Acetic acid (%), Cont1 = Positive control with recommended commercial pectin (%), Cont2 = Negative control without pectin (%).

### 2.4 Jam preparation

Jam preparation was carried out according to the process reported by Islam et al. (2012). Fresh mature strawberries were selected and washed thoroughly with cold water. The washed fruits were cut into pieces with a stainless-steel knife. Pulp was prepared from strawberry fruit by blending the fruit (*Saachi, NL-BL-4361, China*), and the pH was adjusted in the range 2.8–3.3 by addition of 5% acid (*Puro Aceto Vinegar (ZAT), Ethiopia*) (Kopjar*et al.*, 2009). The specified amounts (Table 1) of sugar were mixed with the corresponding quantity of fruit for the jam preparation. The defined amount of jelling agent was mixed with part of the sugar thoroughly. Then the pulp and sugar mix was cooked to boiling. After 15 min, the anchote powder (pectin) with sugar mix was added to the cooking mixture, and the cooking process continued until the jam setting was complete for more than 5 min. Finally, the cooked product was poured into clean, dry sterilized glass jars, and the filled jars were immersed into the boiling water and heated for 10 min. The product then cooled to room temperature and kept for two weeks at the ambient condition to check cross-contamination from mold growth. After two weeks of storage, the samples were subjected to determining the physicochemical parameters and textural property.

### 2.5 Physicochemical analyses

#### 2.5.1 Moisture content (MC)

The moisture content of the jam samples was determined according to the AOAC official method 934.06 (AOAC, 2008).

##### 2.5.1.1 Total soluble solids (TSS)

The total soluble solid content (ºBrix) was determined by direct reading using a hand refractometer (*IP65, Eclipse Range, UK*) at the corrected temperature level.

##### 2.5.1.2 Water activity (a_w_)

The water activity of the Jam was measured at 20 °C using a digital water activity meter (*Novasina, CH-8853 Lachen, LabMaster-aw, Switzerland*).

##### 2.5.1.3 pH

The pH of the jam was measured according to (Bertin et al., 2018) using a pH meter (*pH-016, HINOTEK, China*) after calibrating at room temperature using buffer solutions of pH 4 and 7. Five grams of the jam sample was diluted with 50 ml distilled water to make a 10% (w/v) solution. The solution was stirred for 30 min, and pH measurement was taken with frequent shaking until stable reading.

#### 2.5.2 Titratable acidity (TA)

The titratable acidity of the jam was determined according to a method described by Patil et al. (2013) with slight modification. One gram jam was taken and stirred with 20 mL distilled water, then 2–3 drops of phenolphthalein indicator was added and titrated with 0.1 N NaOH till pink color persisted for 30 s. Finally, TA was determined in terms of the percentage of malic acid as shown in Eq. (1).

(1) $$T_{a}\left(\%\right)=\frac{B\times 0.1\times 0.064\times 100}{W}$$

where T_a_ is titrable acidity in % malic acid, B is the volume of NaOH used (ml); W is the weight of the sample (g), 0.1 is the normality of NaOH solution, and 0.064 is the Milliequivalent factor of malic acid.

### 2.6 Texture analysis

#### 2.6.1 Jam strength

The gel strength of the jam was measured using a texture analyzer (*TA-XT plus, Stable Micro Systems, Surrey, UK*) according to a method described by Santana et al. (2015) with slight modification. A spherical probe (*TA-18B*) was used at a constant 1 mm/s rate until 11 mm penetration depth was reached. The trigger force used was 5 g, with 1.5 mm/s of pre-test speed and 1 mm/s of post-test speed with a return distance of 35 mm. The penetration force was recorded as a function of time. Jam strength was calculated by multiplying the penetration force (g) by the penetration distance (mm).

#### 2.6.2 Texture profile analysis (TPA)

**Figure 2:**
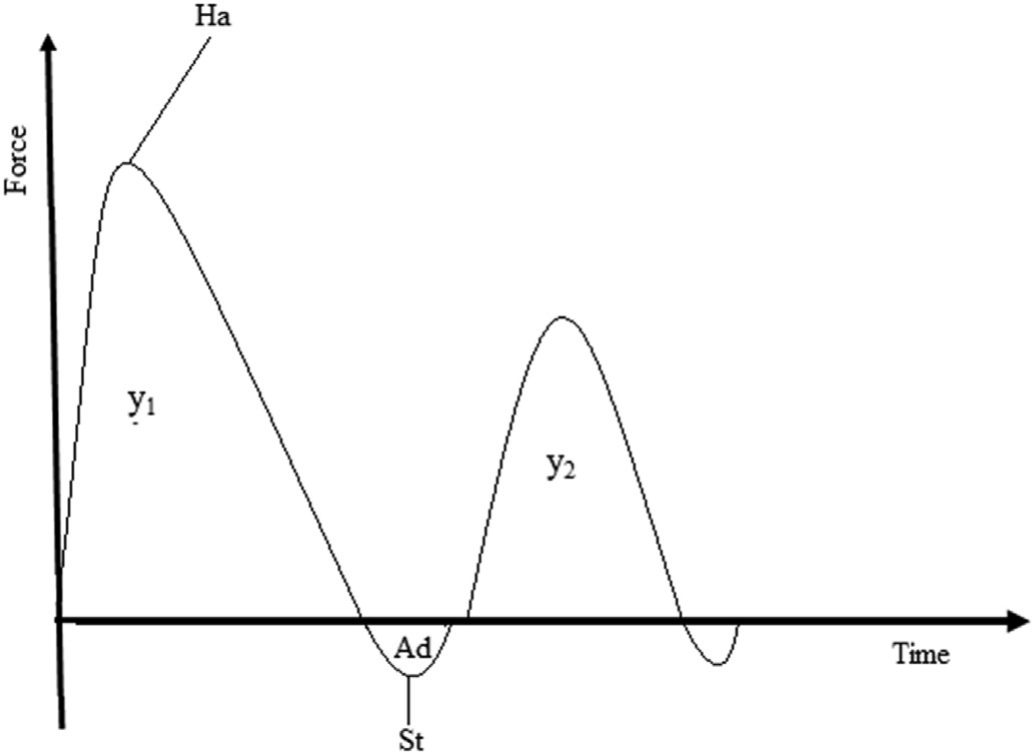
Description of a typical texture profile analysis (TPA) curve (Ha= hardness, y_1_ = area under the 1st peak, y_2_= area under the 2nd peak, Ad= Adhesiveness, St= Stickiness).

Texture profile analysis (TPA) of the jam was carried out using a texture analyzer (*TA-XT plus, Stable Micro Systems, Surrey, UK*) according to the method reported by Santana et al. (2015) and Banaś et al. (2018) with slight modification. The samples were filled to 5 cm height of glass jar sample holder with a diameter of 4.2 cm after being carefully removed from the jars without disturbing their physical integrity. The samples were compressed in two cycles using a spherical probe (*TA-18B*) at a constant rate of 1 mm/s. The trigger force used was 10 g, the pre-test and post-test speeds were set at 3 mm/s, and the return distance was 20 mm. The process of TPA involves dual compression on the sample, imitating two-bites and generating a force-time graph (Figure 2). From the graph, textural parameters such as hardness (Ha), the energy of penetration (EP = y_1_), Adhesiveness (Ad), Stickiness (St), and cohesiveness (Co = y_2_/y_1_) were derived. Hardness was defined by peak positive force, and stickiness was determined from the peak negative force required for the first compression. Cohesiveness was calculated as the ratio of the area under the curve of the second compression to the area under the curve of the first compression. The energy of penetration and adhesiveness were defined by the area under the positive peak and the negative region of the curve, respectively.

### 2.7 Numerical optimization

Numerical optimization of the jams made from anchote powder as a pectin substitute was conducted by setting jam parameters of the positive control sample (0.2% pectin) (*Cont1*) as target values. The control was intentionally used as a standard because the laboratory scale preparation may not match the industrial processing precisely. In addition, complete information (ingredients, processing, and additives) of the commercial jam couldn't be available to set target values from commercially available strawberry jam. Accordingly, the target values of the positive control sample were fixed at the specified values (TSS (61.8 ^o^Brix), pH (2.98), TA (0.73%), a_w_ (0.726), MC (31.61%), GS (450.48 g mm, Ha (31.25 g), EP (459.51 gs), Ad (−64.48 gs), and St (−25.76 g). On the other hand, the independent variables (strawberry fruit, sugar, anchote powder and acetic acid) were considered in the pre-determined ranges as indicated in Table 1. Following the adjustments, the optimized values with a higher desirability were obtained using design expert software (*Design-Expert 13, Stat-Ease, Inc., Minneapolis*).

### 2.8 Data analysis

All the experimental data were subjected to statistical analysis using D-optimal mixture design by Design-Expert 13 software. The statistical significance of each term in the regression equations was evaluated by the two-way ANOVA for each response variable at a significance test level of *p < 0.05*. Appropriate models were selected for each response based on the significance test; and the fitted regression equations for all the parameters. A contour plot for the selected variables was constructed to determine the best mixture composition of the jam ingredients for the responses.

## 3 Results and discussion

### 3.1 Model selection

The ANOVA p-values and selected models for the jam's physicochemical qualities and textural properties are presented in Table 2. Appropriate models for each response variable were selected based on the lowest p-values and the highest adjusted R^2^ value. The result showed the best fit of the special quadratic model to describe changes in TSS; quadratic model for a_w_, MC and cohesiveness; and a linear model for pH and TA of the resulting jam products. On the other hand, the changes in the other texture properties, including gel strength, hardness, energy of penetration, adhesiveness and stickiness, can be better described by a cubic model. The significant (*p < 0.05*) fitting and the non-significant (*p > 0.05*) lack of fit in the selected models confirmed that the models fit well with the tested variables of the strawberry jam prepared using anchote powder as a pectin substitute. All the model terms were significant (*p < 0.05*) for a_w_, pH, TA, gel strength, hardness, and stickiness of the jam. In the models selected for TSS and MC, the interaction term X_1_X_2_ was not significant (*p > 0.05*). Fitting into the cubic model, the term X_1_X_2_ (X_1_ − X_2_) and linear mixture terms were non-significant (*p > 0.05*) for predicting energy of penetration and adhesiveness, respectively. But only the term X_1_X_2_ was significant (*p < 0.05*) in describing the cohesiveness of the resulted jams.

**Table 2:** Analysis of variance (ANOVA) for the jam physicochemical and textural quality variables.

Source	Physicochemical properties					Texture profiles					
	MC	TSS	a_w_	pH	TA	GS	Ha	Co	EP	Ad	St
**Model *(Prob > F)***	Quadratic *(0.0243)*	Special Quartic *(<0.0001)*	Quadratic *(<0.0001)*	Linear *(0.0024)*	Linear *(0.0064)*	Cubic *(<0.0001)*	Cubic *(<0.0001)*	Quadratic *(0.0072)*	Cubic *(0.0049)*	Cubic *(0.0027)*	Cubic *(0.0001)*
**Linear mixture**	0.0162	<0.0001	<0.0001	0.0024	0.0064	0.0063	0.0016	0.0811	0.0259	0.6743	0.0005
**X**_**1**_**X**_**2**_	0.1368	0.5820	0.0002			<0.0001	0.0002	0.0067	0.0169	0.0092	0.0003
**X**_**1**_**X**_**3**_	0.0165	0.0224	0.0041			0.0001	0.0001	0.7633	0.0043	0.0015	<0.0001
**X**_**2**_**X**_**3**_	0.0158	0.0226	0.0045			0.0001	0.0001	0.6960	0.0046	0.0016	<0.0001
**X**_**1**_**X**_**2**_**X**_**3**_						0.0001	0.0001		0.0044	0.0015	<0.0001
**X**_**1**_**X**_**2**_**(X**_**1**_**− X**_**2**_**)**						0.0055	0.0015		0.0525	0.0168	0.0003
**X**_**1**_**X**_**3**_**(X**_**1**_**− X**_**3**_**)**						0.0001	0.0002		0.0049	0.0018	<0.0001
**X**_**2**_**X**_**3**_**(X**_**2**_**− X**_**3**_**)**						0.0002	0.0001		0.0054	0.0018	<0.0001
**X**_**1**_^**2**^**X**_**2**_**X**_**3**_		0.0113									
**X**_**1**_**X**_**2**_^**2**^**X**_**3**_		0.0170									
**X**_**1**_**X**_**2**_**X**_**3**_^**2**^		0.0139									
**Adj. R**^**2**^	0.5978	0.9741	0.9402	0.6065	0.5285	0.9957	0.9927	0.7106	0.9335	0.9511	0.9902
**Lack of fit**	0.3750	0.6439	0.4630	0.6668	0.6054	-	-	0.5999	-	-	-

X_1_ = strawberry fruit pulp, X_2_ = Sugar, X_3_ = Anchote powder, MC = Moisture content, TSS = total soluble solid, a_w_ = water activity, pH = Power of hydrogen, TA = Titrable acidity, JS = Gel strength, Ha = Hardness, Co = Cohesiveness, EP = Energy of Penetration, Ad = Adhesiveness, St = Stickiness, Adj. R^2^ = adjusted coefficient of determination.

The regression equations of the selected models for each response variable were examined to predict the parameters and presented in Table 3. The interaction term of the strawberry fruit and sugar (X_1_X_2_) is excluded from the regression equation developed for TSS and MC; because it is a non-significant (*p > 0.05*) term. For the same reason, the terms X_1_X_3_ and X_2_X_3_ in the regression equation for cohesiveness were also not considered for the predictive model equation. Therefore, the selected model regression equations could be used to predict the corresponding response parameters.

**Table 3:** Regression equation of the selected models for each response examined for the jam quality.

Response Variables	The Selected Model Regression Equation
**Moisture Content**	Y = 32.58 X_1_ + 31.63 X_2_ + 358.02 X_3_ – 360.48 X_1_X_3_ – 366.36 X_2_X_3_
**Total Soluble Solid**	Y = 54.48 X_1_ + 66.02X_2_ + 7921.99X_3_ – 8718.57X_1_X_3_ – 8692.48 X_2_X_3_ + 5274.81X_1_^2^X_2_X_3_ + 4009.31 X_1_X_2_^2^X_3_ – 44680.96 X_1_X_2_X_3_^2^
**Water Activity**	Y = 0.80X_1_ + 0.75X_2_ –8.58X_3_ –0.15X_1_X_2_ +10.32X_1_X_3_ +10.14X_2_X_3_
**pH**	Y = 2.96X_1_ + 3.05X_2_ + 3.73X_3_
**Titrable Acidity**	Y = 0.69X_1_ + 0.60X_2_ + 0.52X_3_
**Gel Strength**	Y = 413.41X_1_ + 230.06X_2_–7.551E + 005X_3_ + 346.70X_1_X_2_ + 1.126E + 006X_1_X_3_ + 1.245E+006X_2_X_3_ – 8.661E+005X_1_X_2_X_3_ – 1472.48X_1_X_2_ (X_1_ – X_2_) – 3.616E + 005X_1_X_3_ (X_1_ – X_3_) – 5.044E + 005X_2_X_3_ (X_2_ – X_3_)
**Hardness**	Y = 39.06X_1_ + 12.69X_2_ –74838.93X_3_ + 18.51X_1_X_2_ + 1.094E + 005X_1_X_3_ + 1.254E + 005X_2_X_3_ –85570.01X_1_X_2_X_3_ – 208.56X_1_X_2_ (X_1_ – X_2_) – 33187.12X_1_X_3_ (X_1_ – X_3_) – 52449.65X_2_X_3_ (X_2_ – X_3_)
**Cohesiveness**	Y = 0.9274 X_1_ + 0.9143X_2_ + 0.9355X_3_ – 0.1245 X_1_X_2_
**Energy of Penetration**	Y = 521.76X_1_ + 327.94X_2_ – 6.897E + 005X_3_ + 136.34X_1_X_2_ + 1.015E + 006X_1_X_3_ + 1.151E + 006X_2_X_3_ – 7.914E + 005X_1_X_2_X_3_ – 1714.12 X_1_X_2_ (X_1_ – X_2_) – 3.141E + 005X_1_X_3_ (X_1_ – X_3_) – 4.775E + 005X_2_X_3_ (X_2_ – X_3_)
**Adhesiveness**	Y = –175.97X_1_ + 103.88X_2_ + 7.999E + 005X_3_ – 141.67X_1_X_2_ – 1.173E + 006X_1_X_3_ – 1.336E + 006X_2_X_3_ + 9.151E + 005X_1_X_2_X_3_ + 2160.24X_1_X_2_ (X_1_ – X_2_) + 3.590E + 005X_1_X_3_ (X_1_ – X_2_) + 5.561E + 005X_2_X_3_ (X_2_X_3_)
**Stickiness**	Y = –50.87X_1_ + 16.51X_2_ + 1.652E + 005X_3_ – 29.16X_1_X_2_ – 2.399E + 005X_1_X_3_ – 2.789E + 005X_2_X_3_ + 1.899E + 005X_1_X_2_X_3_ + 540.97X_1_X_2_ (X_1_ – X_2_) + 71471.19X_1_X_3_ (X_1_ – X_3_) + 1.184E + 005X_2_ X_3_ (X_2_ – X_3_)

Y = Response Variables, X_1_ = strawberry fruit pulp, X_2_ = Sugar, X_3_ = Anchote powder.

### 3.2 Physicochemical properties

#### 3.2.1 Moisture content

The moisture content (MC) of the jam samples (30.7–32.8%) was presented in Table 4. The average MC (31.8%) of the jams was not significantly (*p < 0.05*) different from the MC of both the positive control (31.6%) and the negative control (32.5%). The MC was lowest at minimum fruit pulp proportion but highest with maximum sugar and pectin substitutes. The result lies in the range reported for MC of jams that varied from 28.8 to 35.9% (Garg*et al.*, 2019). It is also in close agreement with MC of strawberry jams; 30.3–31.2% reported by Zubair et al. (2015) and Mohd Naeem et al. (2017). However, the fruit jams' MC was lower than a strawberry jam (48.6%) previously reported (Rodrigues et al., 2017). The ANOVA showed that the interaction of the pectin substitute with both fruit pulp and sugar significantly (*p < 0.05*) affected the MC of the resulting jam (Table 2). The decrease in MC could be due to adding more hydrocolloids (anchote powder) that increases the solid fraction, thus reducing the total moisture (Razak et al., 2018). The MC of foods can be used as an indicator of its shelf life (Mohd Mohd Naeem et al., 2017) so that the lower moisture content indicates that the jams could have a long shelf life. However, higher MC may not necessarily mean free water in food products such as jams since the water is found in bound form with other jam components like sugar and pectin. Razak et al. (2018) stated that water-binding towards the food makes it stable to microbial and chemical deterioration.

**Table 4:** Physicochemical properties of strawberry jams prepared by substitution of pectin with anchote powder.

Treatments	X_1_	X_2_	X_3_	AC	MC	TSS	a_w_	pH	TA
**J1**	45.8	53.0	0.75	0.5	31.64^ab^	61.80^b^	0.749^a^	3.06^a^	0.58^a^
**J2**	45.0	52.8	1.75	0.5	31.14^b^	62.47^b^	0.741^a^	3.13^a^	0.62^a^
**J3**	45.8	53.0	0.75	0.5	31.29^b^	65.37^a^	0.739^a^	3.03^a^	0.64^a^
**J4**	52.5	45.9	1.04	0.5	31.52^ab^	59.30^c^	0.777^a^	2.97^a^	0.64^a^
**J5**	55.0	43.8	0.75	0.5	32.78^a^	55.93^cd^	0.782^a^	2.93^a^	0.72^a^
**J6**	54.8	43.0	1.75	0.5	32.80^a^	50.70^e^	0.801^a^	3.02^a^	0.68^a^
**J7**	55.0	43.8	0.75	0.5	32.08^ab^	54.50^d^	0.795^a^	2.99^a^	0.68^a^
**J8**	47.9	50.5	1.04	0.5	31.67^ab^	62.13^b^	0.754^a^	3.02^a^	0.58^a^
**J9**	45.0	52.8	1.75	0.5	30.74^b^	64.37^ab^	0.738^a^	3.07^a^	0.58^a^
**J10**	54.8	43.0	1.75	0.5	32.13^ab^	50.80^e^	0.799^a^	3.07^a^	0.65^a^
**J11**	49.9	47.9	1.75	0.5	32.34^ab^	58.03^c^	0.736^a^	3.08^a^	0.61^a^
**J12**	52.4	45.5	1.54	0.5	31.02^b^	58.00^c^	0.784^a^	3.00^a^	0.66^a^
**J13**	50.0	48.2	1.33	0.5	31.15^b^	64.03^ab^	0.752^a^	3.09^a^	0.67^a^
**J14**	50.4	48.4	0.75	0.5	32.53^ab^	60.73^bc^	0.741^a^	3.03^a^	0.61^a^
**Cont1**	49.7	49.7	0.20	0.5	31.61^ab^	61.80^b^	0.726^a^	2.98^a^	0.73^a^
**Cont2**	49.8	49.6	0.00	0.5	32.52^ab^	58.83^c^	0.795^a^	3.01^a^	0.73^a^
***Std. Dev.***					*0.43*	*1.50*	*0.01*	*0.03*	*0.03*
***Mean***					*31.77*	*59.58*	*0.76*	*3.04*	*0.64*
***C.V.***					*1.35*	*2.51*	*0.80*	*1.09*	*4.62*

Means that shared the same letters in a column are not significantly different (P < 0.05).

X_1_ = Strawberry pulp, X_2_ = sugar, X_3_ = pectin substitute, AC = Acetic acid, TSS = total soluble solid (°Brix), a_w_ = water activity, MC = Moisture content (%), pH = Power of hydrogen, TA = titrable acidity (%), Cont1 = Control with recommended percent pectin, Cont2 = Control without pectin.

##### 3.2.1.1 Total soluble solids (TSS)

**Figure 3:**
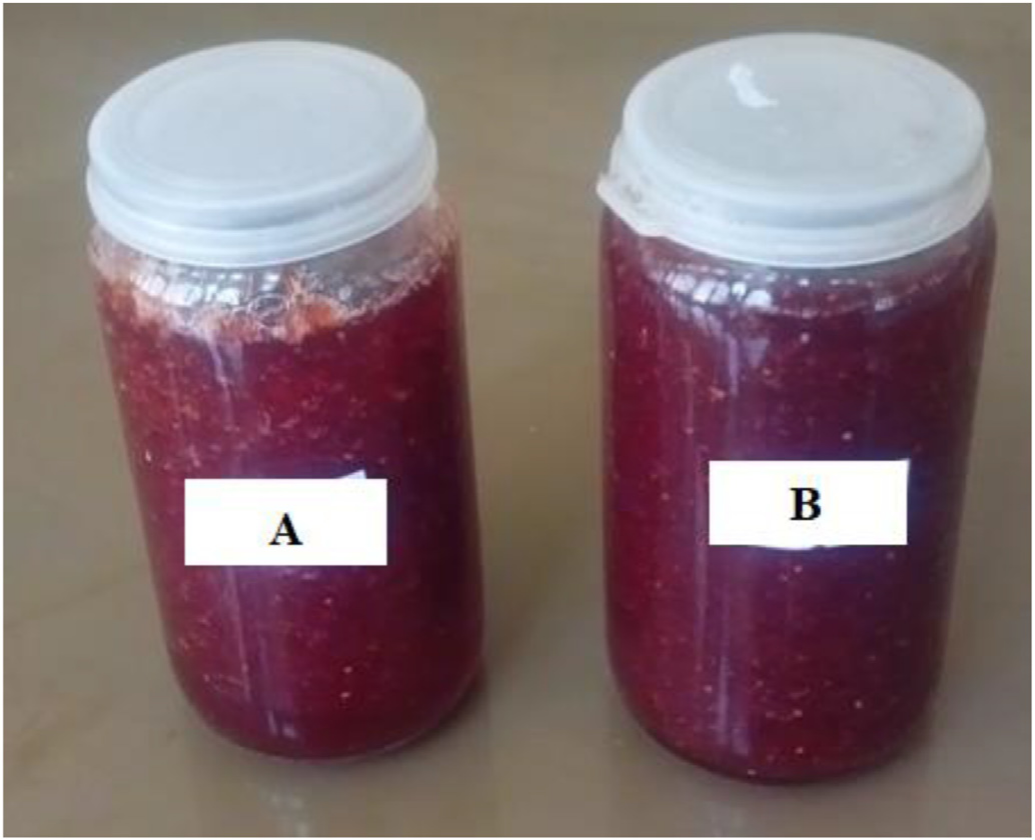
A—The positive control jam prepared with 0.2% pectin, B—the best performing jam product (J_13_) prepared with 1.33% anchote powder.

The TSS of the jam samples was in the range of 50.7–65.4 ºBrix (Table 4), and the linear model terms are significant (*p < 0.05*). All the special quadratic interaction terms were statistically significant (*p < 0.05*), except the interaction of fruit pulp with sugar (X_1_X_2_), to predict the Jam's TSS (Table 2). The TSS value is more altered by the proportion of the commercial sugar than the fruit pulp and anchote powder. This agrees with the increase in the TSS of guava jam by increasing the sugar amount (Nissa et al., 2019); and increasing pectin concentration (Phuong et al., 2016). The highest TSS (65.4 ºBrix) was found in J_3_ prepared at the proportion of 53% sugar, 45.8% fruit, and 0.75% anchote powder (Figure 3). This high TSS value could be because sugar adds more mass to the jam product (Lagha-benamrouche et al., 2018) so that its effect overweighs the effect of the pectin substitute. In agreement with this, Razak et al. (2018) had reported the highest TSS at 0.2% pectin concentration which was studied in the range 0.2–1.0%. The TSS value of J_3_ is greater than the TSS of the positive control (*Cont1*) by 5.8% and the negative control (*Cont2*) by 10.0%. The TSS of some formulations (J_1_, J_2_, J_3_, J_8_, J_9_, J_13_ and J_14_) lays in the ranges of TSS standard set by Codex Alimentarius Commission and the Ethiopian Standards Agency for jams (60–65 ºBrix) (CODEX, 2009; ESA, 2021). The result of this study also agrees with the TSS values reported in jams prepared from Indian blackberry blends (64.33–66.67 ºBrix) (Garg et al., 2019) and bitter jam (59.67–65.33) (Lagha-benamrouche et al., 2018).

##### 3.2.1.2 Water activity (a_w_)

Water activity indicates available free water for microbial growth, chemical and biochemical reactions in foods, and its reduction is important for the extended shelf life of the product. In a jam, a_w_ critically determines the growth of spoilage microorganisms (Sandulachi and Tatarov, 2012). The samples’ a_w_ value in this study ranged from 0.736–0.801 (Table 4). The jam formulations had greater a_w_ than the positive control (0.726) but less than the negative control (0.795), except J_6_ and J_10_ formulations. The result agrees with the documented range (0.75–0.80) for a_w_ levels in jams (Adams and Moss, 2001; NSWFA, 2008). However, Barbosa-Cánovas et al. (2008) had reported higher a_w_ (0.839) in strawberry jam. The results from ANOVA (Table 2) and regression equation (Table 3) showed that a_w_ was negatively affected by anchote powder. The inverse relationship might be attributed to the water-binding minerals and molecules in anchote powder creating more hydrogen bonds formation in acidic conditions resulted in low water activity (Razak et al., 2018). The effect was also reflected in the higher bulk density and water absorption capacity of the blanched anchote tuber flour (Bikila et al., 2022).

##### 3.2.1.3 pH

The pH of the strawberry jam products was in the range 2.93–3.13 (Table 4), and a linear model was statistically the best fit for describing the pH change of the jam samples (Table 2). Most of the jam formulate pH levels were greater than the positive control (2.98). The lowest pH (2.93) was recorded in J_5_ formulation, in which the highest fruit pulp (55%) and lowest anchote powder (0.75%) proportions were used. The highest pH (3.13) was observed in J_2,_ in which the lowest fruit pulp (45%) and highest anchote powder (1.75%) ratios were used. This might be attributed to the presence of organic acids in the fruit pulp (Basu et al., 2014; Kallio et al., 2000). The result showed that the addition of anchote powder did not contribute much to the pH of the resulting jams. The factors exhibited positive coefficients indicating their positive effect on the jams' pH. The lower pH level (higher acidity) of a food product could indicate its longer shelf life. Generally, the pH values were found in the range (2.8–3.5) recommended by Codex Alimentarius Commission (CODEX, 1981). The result also agreed with the pH range (2.92–3.01) reported for blends of Indian blackberry (Garg et al., 2019).

#### 3.2.2 Titratable acidity

Titratable acidity (TA) measures the total acidity value, including concentrations of free protons and undissociated acids present in a solution. The TA of strawberry jam samples prepared using anchote powder as pectin substitute is presented in Table 4 (0.58–0.72%). The highest TA value (higher acidity) was observed in the J_5_ formulation where percent fruit pulp was highest, which could be due to the acidity of the fruit (Basu et al., 2014). This is consistent with the corresponding low pH, and it is not significantly different from the control. The effect of anchote powder on TA was similar to the pH of the jams. The TA is lower compared to that reported in blackberry jam (1.00–1.64%) (Garg et al., 2019) and higher than the acidity recorded in blueberry jam (Pelegrine et al., 2019). But it agrees with that reported for jams produced from dates, orange and apple at different blends (0.6–0.68%) (Olakunle and Aderonke, 2019). The TA of the product may need to be increased to improve its preservation since it is relatively low.

### 3.3 Textural properties

#### 3.3.1 Gel strength of the jam

Gel strength is a measure of the force needed to rupture the jam. Hence higher gel strength will produce a harder jam that reflects its setting quality (Kićanović et al., 2010; Yusof et al., 2019). Depending on various levels of ingredients, the jam samples examined in this study were characterized by average gel strength ranging within 326.39–440.37 g mm (Table 5). Similar findings were reported in strawberry (96.87–317.13 g)**,** gooseberry (106.05–292.65 g) and Indian blackberry (222.29–268.18 g) jams (Banaś et al., 2018; Garg et al., 2019; Korus et al., 2017). The highest gel strength was observed in the J_13_ formulation (*50% fruit, 48.1% sugar and 1.33% anchote powder*), while the lowest was in J_6_ (*54.8% fruit, 43.0% sugar and 1.75% anchote powder*). The increased gel strength with sugar proportion is consistent with the reports of Kićanović et al. (2010) and Lau et al. (2000). Compared to the positive control (*Cont1*), the gel strength of J_13_ is less only by 2.2%; but the gel strength of J_6_ is higher than the negative control (*Cont2*) by 3.5%. The average gel strength of the experimental treatments (322.86 g mm) is lower than the control (*Cont1*) by 17.7%. The variables (strawberry fruit, sugar and anchote powder) significantly (p < 0.05) affected the gel strength of the resulting jam. A similar finding was reported (Kićanović et al., 2010) for different products and sugar types. The result showed that anchote powder had played the greatest role in the gel strength of the resulting strawberry jam at about 1.33%. Similarly, an increase in gel strength of strawberry jams with plant source substitutes (flax seeds and wheat germs) was reported by Korus et al. (2017).

**Table 5:** Texture profile of strawberry jam prepared using anchote powder as pectin substitute.

Treatments	X_1_	X_2_	X_3_	AC	GS (g mm)	Ha (g)	EP (g s)	Co (ratio)	Ad (g s)	St (g)
**J1**	45.8	53.0	0.75	0.5	348.05^j^	27.34^ef^	449.97^f^	0.92^a^	−41.04^ef^	−20.18^d^
**J2**	45.0	52.8	1.75	0.5	357.04^i^	28.77^e^	449.96^f^	0.90^a^	−52.55^d^	−20.77^d^
**J3**	45.8	53.0	0.75	0.5	347.11^k^	27.41^ef^	442.10^g^	0.91^a^	−49.91^de^	−20.41^d^
**J4**	52.5	45.9	1.04	0.5	391.83^f^	30.50^d^	451.87^e^	0.91^a^	−64.57^c^	−19.77^def^
**J5**	55.0	43.8	0.75	0.5	361.76^g^	26.89^ef^	424.01^k^	0.91^a^	−47.53^de^	−18.15^efg^
**J6**	54.8	43.0	1.75	0.5	326.39^o^	26.74^ef^	430.22^i^	0.93^a^	−25.38^f^	−13.78^h^
**J7**	55.0	43.8	0.75	0.5	337.99^m^	26.66^ef^	418.72^l^	0.91^a^	−42.47^e^	−17.55^fg^
**J8**	47.9	50.5	1.04	0.5	342.96^l^	27.26 ^ef^	425.17^j^	0.89^a^	−32.86^f^	−16.64^g^
**J9**	45.0	52.8	1.75	0.5	360.36^h^	28.24^e^	442.94^g^	0.92^a^	−43.01^e^	−21.51^cd^
**J10**	54.8	43.0	1.75	0.5	328.05^n^	26.36^f^	419.13^l^	0.94^a^	−27.16^f^	−14.38h
**J11**	49.9	47.9	1.75	0.5	412.54^d^	32.61^c^	466.39^c^	0.90^a^	−67.51^c^	−23.31^c^
**J12**	52.4	45.5	1.54	0.5	424.15^c^	33.51^b^	480.78^b^	0.90^a^	−86.33^b^	−23.18^c^
**J13**	50.0	48.1	1.33	0.5	440.37^b^	35.09^a^	489.51^a^	0.89^a^	−103.79^a^	−29.22^a^
**J14**	50.4	48.4	0.75	0.5	408.41^e^	30.50^d^	458.94^d^	0.89^a^	−71.46^c^	−24.47b^c^
**Con1**	49.7	49.7	0.20	0.5	450.48^a^	31.25^d^	459.51^d^	0.90^a^	−64.48^c^	−25.76^b^
**Con2**	49.8	49.6	0.00	0.5	314.85^p^	25.83^f^	432.93^h^	0.93^a^	−39.58^ef^	−18.07^efg^
***Std. Dev.***					*2.46*	*0.24*	*5.72*	*0.01*	*4.98*	*0.41*
***Mean***					*370.56*	*29.13*	*446.41*	*0.91*	−*53.97*	−*20.24*
***C.V.***					*0.66*	*0.84*	*1.28*	*0.90*	−*9.23*	−*2.01*

Means that shared the same letters in a column are not significantly different (P < 0.05).

X_1_ = Strawberry pulp, X_2_ = sugar, X_3_ = pectin substitute, AC = Acetic acid, GS = Gel strength, Ha = Hardness, Co=Cohesiveness, EP = Energy of Penetration, Ad = Adhesiveness, St = Stickiness, Cont1 = Control with recommended percent pectin, Cont2 = Control without pectin, Ad = Adhesiveness, St = Stickiness.

#### 3.3.2 Hardness

Hardness (g force) is physically defined as the amount of force needed to achieve a certain level of deformation (Basu and Shivhare, 2010). In the sensorial definition, hardness is the force required to compress food between teeth in the first bite (Nourmohammadi and Ahmadi, 2021). However, in this case, the hardness of the Jam before spread was considered one of the criteria to compare the jam made from anchote powder as a stabilizer substituting commercial pectin with that made from pectin at recommended level (0.2%). Results showed that the hardness of the jam samples ranged from 26.4 to 35.1 g, with the highest value in J_13_ (1.33% anchote powder) formulation and the lowest one is in the J_10_ (Table 5). Basu et al. (2011) stated that hardness increases with the TSS of a jam; the present study also shows a similar trend in the jam hardness and TSS changes. The finding showed that the hardness of the formulations J_11_ (32.6 g), J_12_ (33.5 g), and J_13_ (35.1 g) (*1.75, 1.54, and 1.33% anchote powder proportion, respectively*) was higher than the control product (*Cont1*). The trend shows that there would be the optimum level of the pectin substitute to achieve the desired degree of hardness for the product. The optimum level might be due to a critical level of calcium ion concentration in anchote powder for hardening the gels, below which hardness increases and above which it decreases, as asserted by Lau et al. (2000). This may indicate the contribution of the calcium-rich anchote powder in the formation of a harder gel. Hardness is also among the parameters highly correlated with the spreadability of food products (Ho et al., 2020; Naknaen and Itthisoponkul, 2015; Tifani et al., 2018). Hence, the higher the hardness value of the jam, the higher the force required for the product to spread (Ho et al., 2020). Therefore, J_10_ with low hardness (26.4) might have comparatively the highest spreadability among the samples.

#### 3.3.3 Energy of penetration

The energy of penetration (area under the first pick) refers to the work done for target deformation of the jam (Garg et al., 2019). In the present study, the energy required for penetrating the jam was found in the range of 418.7 and 489.5 g s (Table 5). The highest energy of penetration was observed in the J_13_ formulation prepared from 50.0% strawberry fruit, 48.1% sugar and 1.33% anchote powder. The energy of penetration in the test product formulations J_11_ (466.4 g s) and J_12_ (480.8 g s) were also greater than the control (459.5 g s). The energy of penetration is one of the parameters used to assess the hardness of jam products (Banaś et al., 2018). Hence J_13_ was the hardest among other formulations. The component variables significantly contributed to the energy of jam penetration. Banaś, Korus and Korus (2018) published a similar conclusion in gooseberry jams.

#### 3.3.4 Cohesiveness

Cohesiveness refers to the internal resistance of food structures imparting the ability to combine components of a product, and lower cohesiveness indicates the brittleness of a product (Nourmohammadi and Ahmadi, 2021). The cohesiveness of the strawberry jam prepared with anchote powder varied from 0.89 to 0.94 (Table 5). Most of the results were greater than the control prepared using commercial pectin (0.9). The highest value was recorded in J_10_ formulation, in which the ratio of fruit and anchote powder were higher. These findings suggest that the cohesiveness is more enhanced by the strawberry fruit and anchote powder. The crosslinking of calcium in anchote with carbohydrate and other organic molecules might have been contributed to the slight increase in cohesiveness. But, it was reported that increasing pectin concentration reduced the cohesiveness of jam products (Nourmohammadi and Ahmadi, 2021). From the result, it can be observed that the fruit and sugar interaction (X_1_X_2_) was significant (*p < 0.05*) in the fit model for cohesiveness. The variation could be attributed to the difference in the degree of esterification of the pectin sources (Kopjar et al., 2009). The present finding was consistent with the cohesiveness (0.89) value reported by Ikegaya et al. (2020) in strawberry jam.

#### 3.3.5 Adhesiveness

Adhesiveness corresponds to the total amount of force involved in the withdrawal of the probe from the sample (S. Basu and Shivhare, 2010). In the sensory analysis concept, it is defined as the work required to overcome the gravitational forces between the surface of the jam and the surface of the object that is in contact with the jam (Dias et al., 2018; Nourmohammadi and Ahmadi, 2021). The higher adhesiveness indicates the higher acceptability of the jam product (Teixeira et al., 2020). Adhesiveness could also be perceived as a parameter that reflects the degree of spreadability (Basu et al., 2011). The adhesiveness and spreadability are inversely related parameters (Peinado et al., 2013). In the present study, the adhesiveness of the jam products was varied from −25.4 to −103.8 g s; and the highest was observed in J_13_ jam formulate (Table 5). Compared to the control, the jam formulations J_11_, J_12_, J_13,_ and J_14_ exhibited higher degrees of cohesiveness. The ANOVA results showed that the anchote powder substitute had a positive effect on the degree of adhesiveness of the resulting jam. The effect of increase in the gelling agent concentration agrees with the report of Nourmohammadi and Ahmadi (2021), in which increasing the percentage of pectin increases the adhesiveness of the jam. Similarly, an increase in the percentage of sugar increased the adhesiveness of the jam; and Yang et al. (2021) had reported a similar effect. But the degree of adhesiveness decreased with an increase in the strawberry fruit, which is also consistent with the finding of Nourmohammadi and Ahmadi (2021).

#### 3.3.6 Stickiness

Stickiness (g force) is defined as the maximum force needed to overcome the attractive forces between the surface of the jam and the surface of the probe (Basu and Shivhare, 2010). The stickiness of the jams was varied from −13.8 to −29.2 g, and the highest was observed in J_13_ jam formulate at the proportion of 50% fruit, 48.1% sugar and 1.33% anchote powder (Table 5). Only the stickiness of the same jam formulate was greater than the positive control (−25.8 g). The values do not show a clear systematic trend with change in the concentrations of fruit, sugar, and anchote powder. Basu and Shivhare (2010) had also reported the same observation with pH, pectin, and sugar proportion in mango jam. In addition to hardness and work of adhesion, the stickiness of the jam could also be considered as spreadability parameter (Basu et al., 2011).

### 3.4 Correlation of jam ingredients and measured parameters

The correlation of the jam ingredients with the measured parameters was evaluated using Pearson's correlation coefficient (Table 6). The TSS of the jams was strongly correlated with both the fruit pulp (r = −0.881) and sugar (r = 0.909), confirming that the addition of more sugar increases the TSS. On the contrary, a_w_ was positively correlated with the fruit pulp (r = 0.874) and negatively correlated with sugar (r = −0.878). This relation is because the fruit contains high water, which increases a_w,_ and the sugar adds more solute binding the water, which results in reduced a_w_. For TA, similar associations were observed with the fruit (r = 0.772) and sugar (r = −0.758). But, the correlation of the anchote powder with the other ingredients and the measured variables was low. This observation might be attributed to the relatively low amount of the powder so that its effect could be masked by the major ingredients. The relationship between the texture profile variables measured for the strawberry jam was also evaluated (Table 6). Accordingly, the result shows that the change in gel strength was consistent with the change in energy of penetration (r = 0.909) which shows that the two responses are affected by the factors in a similar fashion. The trend in change of the jam hardness is related to the gel strength (r = 0.972) in line with that reported by Lau et al. (2000) (Table 6). The correlation study also revealed that the increase in adhesiveness was accompanied by an increase in the jam hardness (r = 0.946), consistent with that reported for bilberry jam (Korus et al., 2015). In agreement with reported literature (Basu and Shivhare, 2010; Yusof et al., 2019), the stickiness was affected by the factors in similar trends with adhesiveness (r = 0.915).

**Table 6:** Pearson's correlation coefficients among the measured parameters of strawberry jam substituted with anchote powder.

	X_1_	X_2_	X_3_	TSS	a_w_	pH	TA	GS	Ha	Co	EP	Ad	St
**X**_**1**_	1												
**X**_**2**_	−0.993	1											
**X**_**3**_	−0.027	−0.089	1										
**TSS**	−0.881	0.909	−0.275	1									
**a**_**w**_	0.874	−0.878	0.059	−0.856	1								
**pH**	−0.604	0.536	0.566	0.349	−0.537	1							
**TA**	0.772	−0.758	−0.096	−0.596	0.690	−0.521	1						
**GS**	−0.094	0.084	0.083	0.357	−0.375	0.147	−0.101	1					
**Ha**	−0.058	0.032	0.228	0.285	−0.301	0.229	0.00	0.972	1				
**Co**	0.275	−0.306	0.275	−0.519	0.527	−0.039	0.159	−0.716	−0.610	1			
**EP**	−0.274	0.251	0.186	0.460	−0.438	0.340	−0.131	0.909	0.944	−0.552	1		
**Ad**	−0.053	0.055	0.020	0.370	−0.307	−0.105	0.124	0.938	0.946	0.674	0.910	1	
**St**	−0.368	0.372	0.043	0.623	−0.614	−0.326	−0.153	0.872	0.857	−0.682	0.893	0.915	1

X_1_ = Strawberry Pulp, X_2_ = Sugar, X_3_ = Anchote powder, TSS = total soluble solid, a_w_ = water activity, TA = titrable acidity, GS = Gel strength, Ha = Hardness, Co= Cohesiveness, EP = Energy of Penetration, Ad = Adhesiveness, St = Stickiness.

### 3.5 Numerical optimization

The numerical optimization result for preparing strawberry jam using anchote powder substitute at the targeted values for each parameter gave a blend ratio of 52.4% strawberry fruit pulp, 46.0% table sugar, and 1.07% anchote powder with 0.5% acetic acid (Table 7). The ratio is nearly in agreement with the recommended fruit pulp to sugar ratio for quality jam preparation, where the fruit content is not less than 45% (FAO, 2009). The result showed a reasonably higher degree of desirability score of 0.733 (Table 7). A desirability value of 0.55 was reported in optimizing of cherry jam formulation with stevia (Nourmohammadi and Ahmadi, 2021). The TSS was close to the target value of 61.8 °Brix (less only by 2.9%), and also agrees with the range of 60–65 °Brix for the finished commercial jams, jellies and marmalades as recommended by Codex standard (*Codex Stan 296–2009*) (CODEX, 2009). The TSS could also be increased up to 2 °Brix when stored in a packed glass container at room temperature (Pavlova et al., 2013; Rana et al., 2021). On the other hand, the pH and a_w_ were found in the acceptable range for jam where microbial growth is low (Rawat, 2015). The optimum values of the jam hardness, cohesiveness, and energy of penetration were almost equal to the target values (Table 7). However, a difference of >10% was observed in gel strength, adhesiveness, and stickiness values among the optimized and target values.

**Table 7:** Comparison of the optimal jam quality values prepared using anchote powder with the control.

Physicochemical properties										
Treatments	X_1_	X_2_	X_3_	MC	TSS	a_w_	pH	TA		Desirability
***∗Cont1***	49.7	49.7	0.20	31.61	61.80	0.726	2.98	0.73		0.73
***∗∗Optimized***	52.43	46.00	1.07	31.55	60.01	0.759	3.01	0.66		
***%Difference***	-	-	-	0.19	2.90	-4.55	-1.01	9.59		

**Textural parameters**	**X**_**1**_	**X**_**2**_	**X**_**3**_	**GS**	**Ha**	**Co**	**EP**	**Ad**	**St**	**Desirability**
***∗Cont1***	49.7	49.7	0.20	450.48	31.25	0.90	459.51	−64.48	−25.76	0.73
***∗∗Optimized***	52.43	46.00	1.07	398.49	31.25	0.90	458.13	−71.42	−21.24	
***%Difference***	-	-	-	11.54	0.00	0.00	0.30	−10.76	17.55	

X_1_ = Strawberry Pulp (%),X_2_ = Sugar (%), X_3_ = Pectin (Pectin substitute) (%), MC = Moisture content (%), TSS = total soluble solid (°Brix), a_w_ = water activity, TA = titrable acidity (%), GS = Gel strength (g mm), Ha = Hardness (g), Co = Cohesiveness (ratio), EP = Energy of Penetration (g s), Ad = Adhesiveness (g s), St = Stickiness (g). ∗The control strawberry jam was prepared with 0.2% commercial pectin. ∗∗Values obtained after optimization against the target values for each parameter.

## 4 Conclusion

Strawberry jams formulated with the addition of anchote powder as a pectin substitute were comparable or higher in textural performance and physicochemical quality with the control prepared with the recommended level of commercial pectin. Among the test formulations, J_13_ (*50% strawberry fruit pulp, 48.1% table sugar and 1.33% anchote powder*) formulate jam performed best in most of the measured quality parameters. The optimized result also showed more or less similar results at proportions of 52.43% strawberry fruit, 46.00% table sugar, and 1.07% anchote powder. The finding suggested that the blanched anchote tuber powder can be used in strawberry jam preparation substituting the commercial pectin. However, its compatibility and suitability with other types of jams need further investigation.

## Declarations

### Author contribution statement

Adugna Mosissa Bikila: Performed the experiments; Analyzed and interpreted the data; Wrote the paper.

Yetenayet Bekele Tola: Conceived and designed the experiments; Wrote the paper.

Tarekegn Berhanu Esho, Sirawdink Fikreyesus Forsido: Contributed reagents, materials, analysis tools or data; Wrote the paper.

### Funding statement

This work was supported by College of Agriculture and Veterinary Medicine, Jimma University and Wollega University.

### Data availability statement

Data included in article/supp. material/referenced in article.

### Declaration of interests statement

The authors declare no conflict of interest.

### Additional information

No additional information is available for this paper.
